# Is unplanned extubation avoidable?

**DOI:** 10.1186/cc14700

**Published:** 2015-09-28

**Authors:** Corinne Taniguchi, Carolina SA Azevedo, Cilene Saghabi, Erica A Giovanetti, Guilherme PP de Schettino, Gustavo C da Ferreira, Gustavo FJ de Matos, Karina T Timenetsky, Raquel AC Eid, Ricardo Stus

**Affiliations:** 1 Hospital Israelita Albert Einstein, Morumbi, São Paulo, SP, Brazil

## Introduction

Unplanned extubation (UE) is usually associated with a longer duration of mechanical ventilation (MV), ICU stay and hospitalization. In the Albert Einstein Hospital, it was noticed that there had been an increase in the number of UEs at the beginning of 2014, when protocols such as the daily sedation interruptions were established. UE is more common in patients who are agitated, when they have low levels of sedation or when the endotracheal tube is not well secured.

## Objective

To evaluate an UE prevention program based on five actions.

## Methods

The UE ratio from April to August 2014 was compared with that of the same period from the previous year, comparing the UE index before and after the adoption of a prevention program for patients with a high risk of UE. The prevention of UE was based on five elements: the risk patients were identified during the patient's daily discussion; the endotracheal tube being secured by two different means; arm restraints; the sedation protocol being properly applied; and a sign indicating the potential risk was placed beside the bed.

## Results

During the period April-August 2013 there was a total of 1793 patients on MV against 1720 patients in 2014. During this period, there were 12 UEs in 2013 and eight in 2014 generating an index of 0.7 and 0.5 respectively. In 2014, after the prevention program, there were 33 % less UEs. In 2013 the majority of the UEs occurred with patients who were aware of the intubation and their surroundings (*n *= 8). Within 12 patients, four had UEs owing to poor tube security. Five of those 12 patients were sedated, only one was agitated and two were in the process of spontaneous trials. In point of fact, these two patients did not need any ventilatory assistance after extubation; six of the patients were reintubated and five had to use NIV. In 2014 the majority of the UEs also occurred in patients who were aware of the intubation and their surroundings (*n *= 7) and only one patient had improper tube security. They were all identified as being at a high risk of UE, all restrained, and with double tube security. Three of them were sedated, and were being ventilated in controlled mode. The other five patients were breathing spontaneously (PSV). As the necessity for ventilator intervention after extubation, four of the eight were reintubated; three needed NIV and one needed no support.

## Conclusion

Although this analysis was carried out over a short period of time, the program and the effort of the staff was invaluable in order to diminish and control the number of UEs in our ICU, resulting in a level lower than 3 %.

